# Antimicrobial Susceptibility Profiles of Commensal *Staphylococcus* spp. Isolates from Chickens in Hungarian Poultry Farms Between 2022 and 2023

**DOI:** 10.3390/antibiotics14010103

**Published:** 2025-01-17

**Authors:** Ábel Szabó, Ákos Jerzsele, László Kovács, Ádám Kerek

**Affiliations:** 1 Department of Pharmacology and Toxicology, University of Veterinary Medicine, H-1078 Budapest, Hungary; szabo.abel@student.univet.hu (Á.S.); jerzsele.akos@univet.hu (Á.J.); 2 National Laboratory of Infectious Animal Diseases, Antimicrobial Resistance, Veterinary Public Health and Food Chain Safety, University of Veterinary Medicine, H-1078 Budapest, Hungary; kovacs.laszlo@univet.hu; 3 Department of Animal Hygiene, Herd Health and Mobile Clinic, University of Veterinary Medicine, H-1078 Budapest, Hungary; 4 Poultry-Care Limited Liability Company, H-5052 Újszász, Hungary

**Keywords:** *Staphylococcus*, antimicrobial resistance, minimum inhibitory concentration (MIC), poultry, chickens, Hungary

## Abstract

Background: Antimicrobial resistance is one of the greatest challenges of our time, urging researchers in both veterinary and public health to engage in collaborative efforts, thereby fostering the One Health approach. Infections caused by *Staphylococcus* species can not only lead to significant diseases in poultry but also pose serious threats to human life, particularly in hospital (nosocomial) infections; therefore, it is crucial to identify their antimicrobial resistance. Methods: Our objective was to assess the susceptibility profile of commensal *Staphylococcus aureus* strains (*n* = 227) found in commercial chicken flocks in Hungary through the determination of minimum inhibitory concentration (MIC) values. Results: Based on our findings, resistance to tiamulin (82.8%; 95% CI: 77.4–87.2%) and doxycycline (74.4%; 95% CI: 68.5–79.7%) is the most critical. The 55.1% (95% CI: 48.8–61.3%) resistance rate to enrofloxacin, a critically important antimicrobial, is also concerning. The fact that 58.6% (95% CI: 52.4–64.5%) of the strains were resistant to amoxicillin and 35.7% (95% CI: 29.7–42.1) were resistant to amoxicillin–clavulanic acid suggests that a proportion of the strains produce β-lactamase. Comparing our results with the available human hospital data, it was found that resistance to macrolide antibiotics is similarly high in both cases. Conclusions: Our findings highlight the necessity of conducting regular surveillance studies, which would allow the monitoring of future temporal trends. This information could benefit practitioners making clinical decisions to successfully treat infections. To uncover the underlying causes of multidrug resistance, next-generation sequencing can be employed to elucidate the genetic basis of phenotypic resistance.

## 1. Introduction

Antimicrobial resistance (AMR) is a global public health challenge that is receiving increasing attention. Following the adoption of a new strategy in 2020, the veterinary profession continues to play a critical role in combating AMR [1]. Inappropriate use of antibiotics in recent decades, along with social and economic trends, have significantly accelerated the selection and spread of resistant bacteria, leading to a marked increase in resistance-related mortality [2]. Even the most conservative estimates suggest that by 2050, the number of deaths attributable to AMR could reach 10 million annually if antibiotic use continues at the current rate and advancements in therapeutic approaches and active substances do not keep pace [3]. While the significance of this issue has been widely recognized, the spread of multidrug-resistant bacteria and the resulting infections continue to rise [4].

Poultry are one of the most widely raised food-producing animals worldwide, with chicken being the most commonly farmed species, with over 100 billion tons of chicken meat produced globally each year [5]. The primary reasons for this are the low production costs and the lack of cultural and religious restrictions on its consumption [6]. In poultry, infections caused by avian pathogenic bacterial species pose significant health challenges, threatening animal welfare, productivity, and the effectiveness of antibiotic treatments [7,8,9]. Resistant bacteria originating from animals can infect humans through direct contact or via animal-derived food products [10,11,12], and this is particularly true in the poultry industry [13].

The poultry industry is the second largest consumer of antibiotics after the pig industry [14], making it particularly important to reduce or replace antibiotics in these sectors [15]. It is also important to note that antibiotic usage exerts a significant influence on the gut microbiome, thereby shaping the resistome and its dynamics [16], particularly when bacteria are exposed to sublethal injuries, which is especially true for *Escherichia coli* [17,18]. Several studies have observed antibacterial effects using plant essential oils [19,20], plant extracts [21,22,23,24,25], and antimicrobial peptides [26]. Propolis, whose composition significantly influences its antimicrobial efficacy, is also a promising natural substance [27,28,29]. Maintaining effectiveness can also be supported by selecting treatments for infectious diseases based on pharmacokinetic/pharmacodynamic studies [30,31], and appropriate preventive disease control measures are equally important [32,33].

In addition to the harm it causes on commercial poultry farms, *Staphylococcus aureus* (*S. aureus*) poses a threat to public health due to widespread antimicrobial resistance [34,35,36]. Animal infections not only cause issues within veterinary medicine but also play a role in the transmission of pathogens from animals to humans [37]. *Staphylococcus* species are Gram-positive bacteria with a wide host range, commonly inhabiting the skin, mucous membranes, and respiratory tracts of both humans and birds [38,39]. Among them, *S. aureus* stands out as the most pathogenic, causing an extensive spectrum of diseases, ranging from superficial skin infections to life-threatening conditions such as toxic shock syndrome and sepsis [40,41,42]. While the carriage rate of *S. aureus* in humans is around 20–30%, this rate is as high as 90% in poultry [43,44]. Numerous studies have reported on the zoonotic transmission of methicillin-resistant *S. aureus* (MRSA) from food-producing animals to humans [34,36,45,46], with these strains being detected in both healthy and diseased poultry in some cases [47,48]. The proportion of MRSA infections has significantly increased worldwide from the late 1980s to 2000 [35], and the epidemiological classification of human infections is divided into healthcare-associated and community-associated categories [49]. The list of agents available for the successful treatment of MRSA is limited; however, trifluoperazine shows promising results [50].

The widespread occurrence of MRSA strains detected in chicken meat has led to an antibiotic resistance crisis worldwide [51]. These strains have been proven to contaminate human food, causing staphylococcal food poisoning [52]. Recently, there have been several outbreaks linked to mass foodborne infections [53]. Chicken meat can be a potential source of zoonotic MRSA infection, and the consumption of contaminated food has been shown to result in colonization in humans [54,55]. *S. aureus* is frequently found on the surface of poultry meat and plays a significant role in the spread of antimicrobial resistance [56]. It has also been confirmed that resistance can easily develop against vancomycin, a drug of critical importance to public health [57].

Staphylococcal food poisoning is associated with symptoms such as vomiting, septicemia, pneumonia, and toxic shock syndrome [58], with the enterotoxins remaining in the food even after the meat has been heat-treated [52]. These enterotoxins form a superfamily of small-molecular-weight pyrogenic exotoxins that elicit a strong antigenic response. They disrupt adaptive immunity by stimulating T cells, which induce inflammatory cytokine production. The genes encoding these toxins are typically found on mobile genetic elements (MGEs), contributing to their widespread dissemination [58].

Additionally, *S. aureus* is capable of producing biofilms, making contaminated surfaces in meat processing facilities a continuous source of contamination for meat products [59,60,61]. These biofilm formations involve adhesive and matrix molecules that sense bacterial surface components, with biofilm maturation occurring through the expression of polysaccharide adhesion molecules encoded by the intracellular adhesion gene cluster operon [62,63].

In Europe, the incidence of MRSA infections has decreased in recent years, although significant geographical variations remain, with a prevalence of 1% in Northern Europe and up to 50% in Southern Europe [64]. In the USA, a 17% reduction in bloodstream infection cases was observed between 2005 and 2016. However, it is important to note that morbidity remained high, with 119,247 cases, and mortality accounted for 19,832 deaths [65].

Due to its public health significance, our objective was to assess the susceptibility profile of commensal *S. aureus* strains occurring in commercial chicken flocks in Hungary. AMR represents a global challenge that demands a comprehensive and coordinated strategy to preserve the effectiveness of antibiotics. Addressing this issue requires collective effort, collaboration, and a commitment to responsible practices.

## 2. Results

### 2.1. Regional Distribution and Origin of the Collected Samples

A total of 227 strains were isolated from 23 commercial chicken farms. Of these, eight were from layer flocks, seven from breeding flocks, and eight from broiler chicken farms. The majority of the isolates (97.8%) were respiratory swabs, while 2.2% were cloacal swabs. The prevalence, based on the number of samples and isolates, was 32.9%. The 95% confidence interval (CI) was between 29.5% and 36.5%, calculated from the 690 samples analyzed. The isolates were almost evenly distributed across Hungary’s seven regions, with the highest number of isolates (18.1%) coming from the Dél-Alföld region. The samples originated from at least three farms representative of each region to strive for nearly representative sampling. Of the isolates, 35.7% were from broiler chickens, 26.9% from breeding flocks, and 37.4% from layer flocks. In terms of age distribution, 35.7% of the isolates were from younger than six weeks, and 64.3% were from adult birds. Regarding flock size, 53.3% of the isolates came from small farms (5001–50,000 birds), 24.3% from medium-sized farms (50,001–100,000 birds), and 22.4% from large farms (>100,001 birds).

### 2.2. Antimicrobial Susceptibility Testing

We conducted susceptibility testing for a total of 15 antibiotics of veterinary and public health significance. For nine of these antibiotics, clinical breakpoints were available, allowing us to determine the proportion of resistant strains.

**Figure 1:**
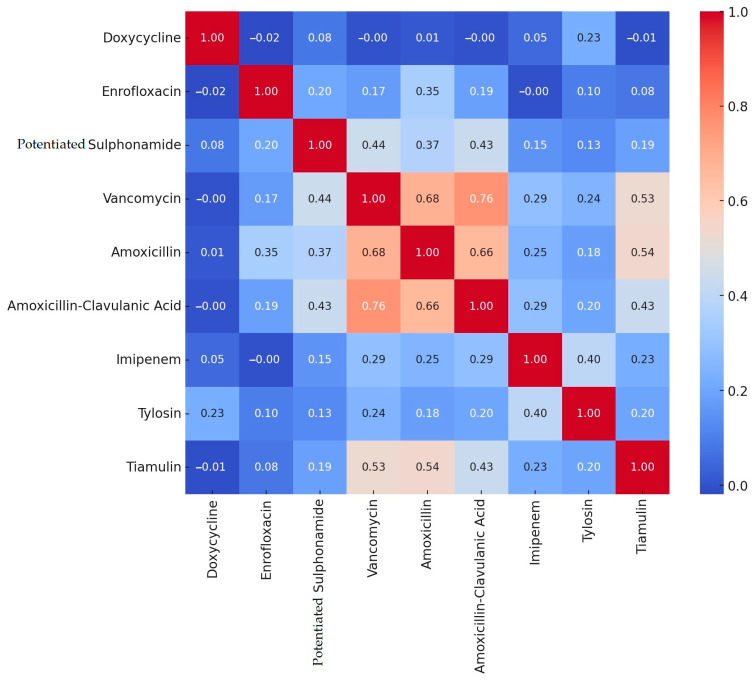
Correlation analysis of resistance between different active substances in *Staphylococcus* strains isolated from domestic chickens (*n* = 227).

Based on the resistance levels determined by clinical breakpoints, we performed a correlation analysis among the different active substances (Figure 1).

During the correlation analysis, we found strong positive correlations between amoxicillin–clavulanic acid and vancomycin (0.76), between amoxicillin and vancomycin (0.68), between amoxicillin and amoxicillin–clavulanic acid (0.66), as well as between vancomycin and tiamulin (0.53), and between amoxicillin and tiamulin (0.54). Negative correlations were negligible.

**Figure 2:**
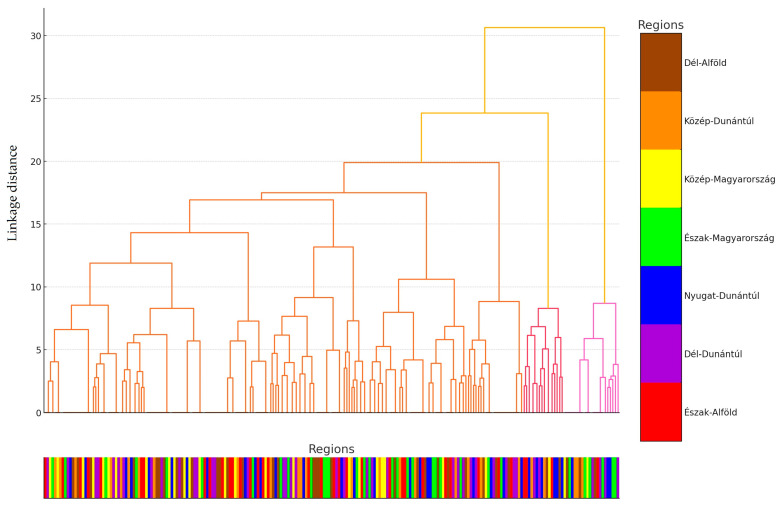
Cluster analysis of *Staphylococcus* strains isolated from domestic chickens (*n* = 227) based on internal homogeneity and external heterogeneity of the data. For better clarity, the data points were assigned to their respective regional origins and color-coded accordingly, with the appropriate color assigned below the horizontal axis for scaling.

We performed cluster analysis on the data (Figure 2). The hierarchical cluster analysis grouped the isolates into three main clusters, which were differentiated based on linkage distance. The clusters displayed geographic patterns, visualized using color codes. A visual inspection of the dendrogram suggests that isolates from the same region often belonged to the same cluster, particularly in the case of the Dél-Alföld and Észak-Magyarország regions.

The largest distance between clusters was observed for isolates from Közép-Magyarország and Nyugat-Dunántúl. These geographically driven groupings may indicate that differing antibiotic usage practices, livestock management approaches, or environmental factors across regions influence the resistance profiles of the isolates.

These findings emphasize the importance of region-specific monitoring programs and the need for antimicrobial resistance strategies tailored to local contexts. A detailed analysis of the dendrogram provides an opportunity to identify subclusters and investigate the similarities and differences between the groups.

**Figure 3:**
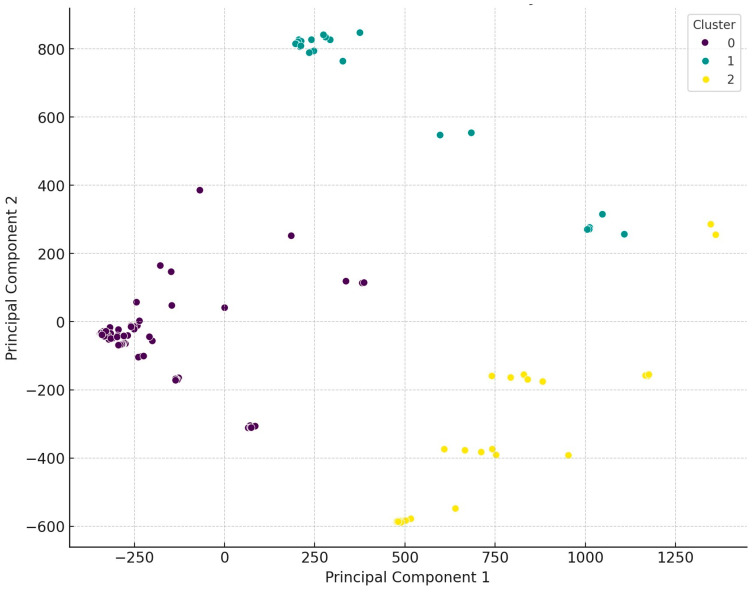
Visual representation following the principal cluster analysis of *Staphylococcus* isolates from domestic chickens (*n* = 227). The data were classified into three main clusters, which are clearly distinct from one another.

Using the dendrogram, we identified three main clusters and then performed principal component analysis (PCA) to visualize the differences between the clusters (Figure 3). PCA is a statistical method used to reduce the dimensionality of data while preserving as much variance as possible. The analysis identifies new axes (principal components) along which the variance is maximized. The first principal component explains the largest portion of the data’s variance, the second principal component explains the next largest portion of the remaining variance, and so on.

Regarding the distribution of isolates across the three main clusters, 67.4% belonged to the first cluster (purple), which showed an even regional distribution, except for the Észak-Alföld region, where significantly fewer isolates were collected. The second cluster (green) comprised 14.9% of the isolates, with dominance in the Dél-Alföld and Dél-Dunántúl regions, while no isolates from this cluster were found in the Nyugat-Dunántúl and Észak-Magyarország regions. The third cluster (yellow) included 17.6% of the isolates, with no isolates from the Észak-Magyarország region, only one sample from Közép-Magyarország, and four isolates from Dél-Dunántúl. The distribution of isolates was even across the other regions.

We examined whether there was a significant difference in resistance based on the source of the isolates (respiratory tract, cloaca) (Table 1). Although, the number of strains isolated from cloacal isolates was low, a significant difference was observed for only one active substance, doxycycline (*p* > 0.0001). This indicates that resistance levels for this antibiotic vary markedly between these sampling sites. This discrepancy may reflect site-specific selective pressures, such as localized antibiotic exposure or microbiota adaptations, underscoring the importance of tailoring treatment strategies to the infection’s location. For other antibiotics tested, including enrofloxacin and amoxicillin, no significant differences were observed (*p* > 0.05), suggesting a relatively uniform resistance profile across anatomical sites. These findings emphasize the need for targeted surveillance of resistance trends while reinforcing the importance of prudent antibiotic use in both respiratory and enteric infections.

In terms of utilization types (Table 2), significant differences in the level of resistance were observed only in a few cases. Between laying and broiler flocks, differences were noted for doxycycline (*p* = 0.0336), potentiated sulfonamide (*p* = 0.0074), and tylosin (*p* = 0.0077). For laying and breeding flocks, significant differences were observed for enrofloxacin (*p* = 0.0402) and potentiated sulfonamide (*p* = 0.0109). When comparing broiler and breeding flocks, significant differences were found for imipenem (*p* = 0.0233) and tylosin (*p* = 0.0291). These findings highlight the variability in resistance profiles across poultry production types, likely influenced by differences in antibiotic use practices and management systems, warranting targeted interventions to optimize antibiotic stewardship in each production type.

Comparing the differences between age groups (Table 3), significant differences in resistance levels were observed for doxycycline (*p* = 0.058), imipenem (*p* = 0.0124), and tylosin (*p* = 0.0026). These findings suggest that age-related factors, potentially including differences in antibiotic exposure, immune system development, and management practices, may influence resistance patterns. Addressing these age-specific disparities could enhance antibiotic stewardship strategies and reduce the risk of resistance development across poultry populations.

When considering flock size (Table 4), significant differences were observed between small (5001–50,000) and medium (50,001–100,000) flocks for vancomycin (*p* = 0.0012), amoxicillin (*p* = 0.0392), and tiamulin (*p* > 0.0001). Comparing small and large (>100,001) flocks, flock size had a more pronounced impact on resistance levels for a greater number of active substances. However, between medium and large flocks, there were no significant differences except for doxycycline (*p* = 0.0196) and potentiated sulfonamide (*p* = 0.0006). These findings highlight the role of farm size in influencing resistance patterns, likely driven by variations in antibiotic usage, stocking densities, and biosecurity measures. Tailored interventions targeting larger farms may be critical in mitigating antimicrobial resistance in poultry production systems.

From the determined MIC values, a frequency table was created for each active substance (Table 5). Both the MIC_50_ and MIC_90_ values remained below the clinical breakpoints for imipenem and vancomycin. Only the MIC_50_ value remained below the breakpoint for amoxicillin–clavulanic acid, tylosin, and potentiated sulfonamide. MIC_50_ and MIC_90_ are two important metrics used to evaluate MIC tests in determining the effectiveness of antimicrobial agents at a population level. These metrics indicate the effectiveness of different concentrations of antimicrobial agents against microorganisms. The MIC_50_ is the lowest concentration of the antimicrobial agent that inhibits the growth of 50% of the microorganisms tested. It provides a median value for the susceptibility of the population and is often used to assess the general resistance profile of the population. The MIC_90_ is the lowest concentration of the antimicrobial agent that inhibits the growth of 90% of the microorganisms tested. It is a more conservative measure of the resistance distribution within the population, indicating the concentration needed to effectively inhibit the majority of the population.

When compared to the ECOFF value, the MIC_50_ values for tylosin and vancomycin were the only ones that remained below this threshold. The ECOFF is a value used in the epidemiological study of antimicrobial resistance to distinguish between wild-type microorganisms and resistant strains. It is based on the distribution of MIC values and represents the highest MIC value at which microorganisms can still be considered wild-type. Below this value, the natural population of microorganisms is sensitive to the antimicrobial agent, and no acquired resistance is present. These are strains in which no detectable acquired resistance mechanism against the antimicrobial agent is present. Although the ECOFF cannot be used as a clinical susceptibility breakpoint on its own, it provides valuable information for understanding the epidemiology of resistance, supporting treatment decisions.

The frequency of MIC values for active substances without clinical breakpoints is summarized in Supplementary Table S1.

**Figure 4:**
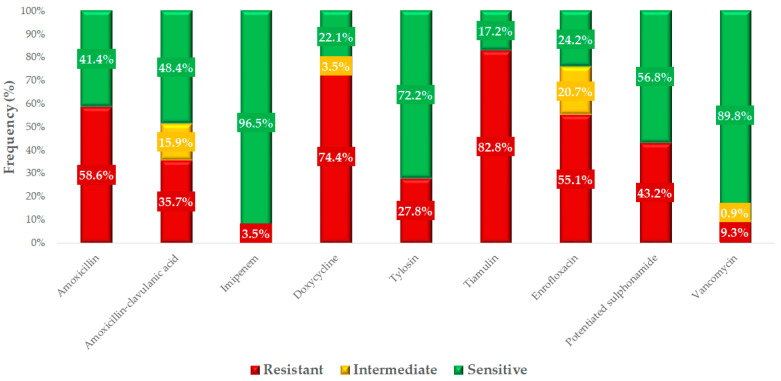
Resistance profile of commensal *Staphylococcus* strains (*n* = 227) isolated from domestic chickens against antibiotics of veterinary and public health significance.

Based on the clinical breakpoints, we determined the resistance profile for each active substance (Figure 4). The highest levels of resistance were observed for tiamulin (82.8%, 95% CI: 77.4–87.2%) and doxycycline (74.4%, 95% CI: 68.5–79.7%). The low resistance levels observed for imipenem (3.5%, 95% CI: 1.8–6.8%) and vancomycin (9.3%, 95% CI: 6.1–13.7%) are considered minimal. The difference in resistance levels between amoxicillin (58.6%, 95% CI: 52.4–64.5%) and amoxicillin–clavulanic acid (35.7%, 95% CI: 29.7–42.1%) suggests that a proportion of the strains are β-lactamase producers.

**Figure 5:**
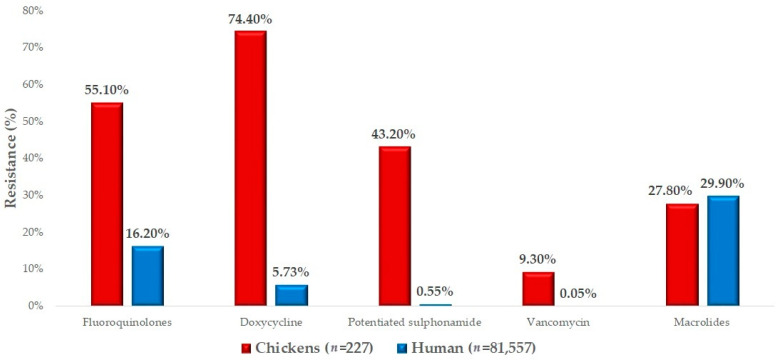
Comparison of isolates isolated from domestic chickens with available human resistance data for *Staphylococcus* isolates.

We had the opportunity to compare our results with human resistance data (Figure 5). The resistance levels for macrolides were very similar between the isolates isolated from domestic chickens and the results of susceptibility tests from human isolates. In the case of fluoroquinolones, the resistance level was significantly higher in veterinary isolates (55.1%, 95% CI: 48.8–61.3%) compared to human health data (16.2%, 95% CI: 15.9–16.5%). For doxycycline, we observed an extremely high resistance rate of 74.4% (95% CI: 68.5–79.7%), compared to only 5.7% (95% CI: 5.6–5.9%) in human isolates. A similar difference was observed for potentiated sulfonamides. The proportion of strains resistant to vancomycin, while lower overall, was also much higher in chickens (9.3%, 95% CI: 6.1–13.7%) compared to the human resistance rate of 0.05% (95% CI: 0.04–0.07%).

## 3. Discussion

In this study, a total of 227 commensal *Staphylococcus* isolates from 23 large-scale chicken flocks were subjected to susceptibility testing. Our results were largely in line with the existing literature, although the findings of different studies do vary widely. The observed differences in results are likely primarily due to variations in antibiotic usage practices across countries, particularly considering the significant discrepancies in legal regulations between European Union and non-EU countries. Our findings also highlighted differences between utilization types, which play a key role in antibiotic selection. Numerous other factors can influence the resistance patterns of a given country or even a specific region. However, it is important to emphasize the undeniable role of commensal strains in maintaining resistance.

We observed a resistance rate of 58.6% for amoxicillin, which is in line with the findings reported in the literature; Kim et al. reported 51.2% resistance to penicillins [66], Abunna et al. reported 65.6% [67], and Rafiq et al. observed 85.4% resistance [68]. The similarity of the results supports the relevance of our study, while also highlighting that the global presence and high prevalence of amoxicillin resistance remain a significant challenge, particularly at the intersection of animal and human health.

For doxycycline, the resistance rate was 74.4%, with Miranda et al. reporting a similar rate of 58.4% [69], Boamah et al. reporting 43.8% [70], Rafiq et al. reporting 55.6% resistance [68], and Kim et al. observing 38.8% resistance to tetracycline [66]. This discrepancy is likely explained by the widespread and prolonged use of doxycycline, particularly in poultry farming, where the antibiotic has historically been employed both for preventive and therapeutic purposes. Furthermore, variations in resistance patterns observed across different geographical regions are likely influenced by national regulations, usage practices, and the intensity and methods of livestock management.

The resistance rate for enrofloxacin in our tests was 55.1%, similar to Rafiq et al.’s finding of 57.9% [68]. However, Abunna et al. reported a resistance rate of only 4.7% [67], Sonola et al. observed 3.7% [71], and Boamah et al. reported just 1.9% resistance to enrofloxacin [70]. Kim et al. found 33.9% resistance to ciprofloxacin [66]. The differing resistance rates are likely influenced by geographical variations, differences in antibiotic usage practices, and regulatory frameworks. Enrofloxacin continues to be widely used in the poultry industry, which may contribute to higher resistance rates. Given that it is a critically important antibiotic reserved for hospitalized human patients, its use must be significantly reduced, and efforts in this direction are already underway. Monitoring ciprofloxacin resistance levels is particularly important, as it is a critical drug in human medicine. Moreover, a portion of enrofloxacin is metabolized into ciprofloxacin in animal bodies, potentially contributing to resistance development. These findings underscore the necessity of responsible fluoroquinolone use in both veterinary and public health sectors.

For vancomycin, we observed a resistance rate of 9.3%; Mkize et al. found 14% resistance in strains isolated from fecal isolates, but 61.9% resistance in abattoir isolates [72]. Abunna et al. reported 59.4% resistance [67], while neither Benrabia et al. [73] nor Lin et al. [74] found any resistant strains in MRSA isolates. The observed differences are likely due to the variation in sampling sources, such as abattoir samples, as well as differences in antibiotic usage practices and local antimicrobial regulations. The low resistance rate observed in our study may indicate that vancomycin use has been strictly limited in veterinary practice, as it has never been approved for use in poultry and is now entirely banned in animal health due to its critical importance as a lifesaving, last-resort antibiotic in human medicine. However, there was a period when avoparcin, a related compound within the same class, was permitted for use in poultry, which may have contributed to the dissemination of environmental resistance. These findings underscore the importance of continuous monitoring of vancomycin resistance, especially given its vital role in the treatment of MDR infections in human healthcare.

For amoxicillin–clavulanic acid, our experiments showed a resistance rate of 35.7%, whilst Benrabia et al. observed 100% resistance in MRSA strains [73]. Sonola et al. reported 9.1% resistance [71], and Boamah et al. did not find any resistant strains [70]. The varying resistance rates to amoxicillin–clavulanic acid can likely be attributed to factors such as differences in sample sources, variations in antibiotic usage practices, and disparities in regulatory frameworks across different countries. The moderate resistance rate observed in our study may be due to the fact that the use of this antibiotic combination is not authorized in the poultry sector. However, the extremely high resistance rates reported, particularly among MRSA strains, underscore the potential presence of resistance mechanisms. These findings highlight the importance of ongoing monitoring and careful regulation of this critical antibiotic combination, especially given its significance in human healthcare.

For imipenem, we found a resistance rate of 3.5%, while Moawad et al. reported 12.8% resistance [75]. For tylosin, we observed a resistance rate of 27.8%, and Lin et al. reported 43% resistance [74]. For tiamulin, we found 82.8% resistance, compared to 100% resistance in Nemeghaire et al.’s study [76]. The low resistance rates observed for imipenem are consistent with its designation as an antibiotic reserved exclusively for human healthcare. The moderate resistance to tylosin may reflect its extensive use in the poultry industry, particularly as a macrolide antibiotic commonly employed to treat respiratory infections. However, the high resistance rates observed for tiamulin are concerning and likely stem from its long-term and intensive application. These findings underscore the critical need for responsible antibiotic use regulations. Further investigation into resistance patterns, particularly the genetic mechanisms underlying these variations, is essential for developing targeted preventive measures and treatment strategies.

For potentiated sulfonamide, we observed a resistance rate of 43.2%. However, results from other studies varied, with Abunna et al. reporting 14.1% resistance [67]; Benrabia et al. reporting 27.8% resistance in breeding flocks, 27.8% in laying hens, and 26.3% in broiler chickens [73]; Rafiq et al. reporting 50.5% [68]; and Boamah et al. reporting only 5.5% resistance [70]. The observed resistance rate of 43.2% against potentiated sulfonamides in our study can be considered moderate; however, the literature shows significant variability. These differences are likely attributable to the geographic origins of the samples, variations in antibiotic usage practices, and differences in the study populations and methodologies employed. The role of potentiated sulfonamides in veterinary medicine, particularly for the treatment of respiratory and gastrointestinal infections, may contribute to the development of resistance. These findings highlight the need for more prudent regulation of this antibiotic class and further investigation into the mechanisms sustaining resistance.

Our studies revealed that the type of utilization significantly influenced the level of resistance, with the most notable differences observed between broiler and breeding flocks. The age of the flocks (juvenile vs. adult) also significantly impacted resistance levels for several active substances. However, the most significant differences were related to flock size, with small (5001–50,000) and large (>100,001) flocks showing significant differences in resistance for five out of nine active substances. Previous studies have shown that similar differences exist in chickens based on utilization type [77]. The observed differences suggest that various types of production systems employ antibiotics with differing intensities and spectra. Broiler flocks, primarily raised for meat production, are likely exposed to more frequent and broader-spectrum antibiotic use, potentially resulting in higher resistance levels. The significant impact of age (young vs. adult) on resistance levels indicates that developmental stages and immune status play a critical role in shaping the microbial ecosystem. Younger animals are more susceptible to infections, leading to more frequent antibiotic use and an increased risk of resistance development. Monitoring age-specific resistance trends can aid in developing more targeted antibiotic usage strategies. The differences between flock size and resistance levels highlight that density and production scale significantly influence the development of AMR. Larger flocks, with more intensive rearing conditions and faster bacterial spread, may experience higher selective pressure. In contrast, smaller flocks, with differing management and biosecurity practices, may exhibit distinct resistance dynamics.

Comparing our results with available human resistance data, we found a 55.1% resistance rate to fluoroquinolones in chickens, compared to 16.2% in humans. Sonola et al. reported 11.7% resistance to ciprofloxacin in human isolates [64]. The significantly higher fluoroquinolone resistance observed in the poultry sector highlights the potential risk posed by veterinary resistance levels to human health. In our studies, we observed 27.8% resistance to tylosin, while the human resistance rate was 29.9%. Sonola et al. reported 62.8% resistance to macrolides [64]. For doxycycline, we observed a resistance rate of 74.4% in chickens, while the human data showed only 5.7% resistance. Nazarchuk et al. reported 34.6% resistance in strains isolated from human hospitals [78]. The significantly higher resistance rates observed in animals, particularly in the poultry sector, compared to those measured in humans highlight the urgent need for coordinated actions to address AMR. Resistance to critical antibiotics such as fluoroquinolones poses a serious risk in terms of zoonotic transmission and the potential for resistance genes to spread across species.

The resistant strains identified in our study hold dual significance for public health. While they could directly threaten human health, especially in immunocompromised individuals or those with disrupted microbiomes, their primary concern lies in their role as reservoirs of resistance genes. These genes, capable of horizontal transfer to pathogenic bacteria, present a critical zoonotic risk, especially when transmitted via the food chain through inadequately processed animal-derived products. Understanding these mechanisms and mitigating associated risks remain urgent priorities.

The extensive use of antibiotics in poultry farming is a key driver of resistance emergence. Targeted interventions, including reducing antibiotic use, promoting alternatives such as probiotics or phytogenics, and enforcing stricter antimicrobial regulations, are essential. Enhanced biosecurity measures and proper poultry product handling can further minimize transmission risks. These efforts align with the One Health framework, addressing the interconnectedness of human, animal, and environmental health.

Future research should prioritize studying horizontal gene transfer mechanisms and the role of commensal *Staphylococcus* as resistance reservoirs, which may also be associated with wild birds [79]. Investigating antibiotic alternatives and optimizing usage practices will be critical, alongside regular resistance monitoring and improved biosecurity measures. Collaborative efforts between veterinary and public health sectors, bolstered by clear regulations and awareness campaigns, are vital to curb antimicrobial resistance and safeguard the efficacy of antibiotics. Routine surveillance studies, like ours, provide valuable data that reinforce the necessity of a unified One Health approach to combat antimicrobial resistance.

## 4. Materials and Methods

### 4.1. Origin of Strains and Human Data

The examined strains were collected between 2022 and 2023 during routine diagnostic investigations performed by veterinarians serving large-scale livestock farms in collaboration with poultry health experts of the Department of Animal Hygiene, Herd Health and Mobile Clinic. A total of 690 samples were collected from 23 commercial chicken farms selected randomly from all over Hungary. Information available for the samples included the organ (trachea, cloaca), the type of bird (meat, eggs, breeding), the age (young, adult), and the flock size (5001–50,000; 50,001–100,000; >100,001), and based on the location of the flock, the samples were classified into seven administrative regions of Hungary. The sampling process was guided by specific criteria, including comprehensive coverage of Hungary’s geographical regions and voluntary participation. Veterinarians routinely collect cloacal and respiratory samples from live animals during diagnostic procedures. The samples were collected using Amies transport swabs (sterile, without charcoal, and equipped with standard aluminum shafts (Biolab Zrt., Budapest, Hungary)). For each animal, two samples were taken: an oropharyngeal swab from the area near the tracheal entrance and a cloacal swab. The sampling procedure involved rotating the swab 3 to 5 times in a circular pattern. The samples were then transported in the provided media to the reference laboratory under controlled conditions at 2–8 °C, from where they were subsequently forwarded to us for further analysis. For the isolation of *Staphylococcus* strains, the samples were streaked onto CHROMagar™ *Staph aureus* agar (Chebio Fejlesztő Kft., Budapest, Hungary). The isolates were further processed after collection, and the pure cultures were frozen in the Microbank™ system (Pro-Lab Diagnostics, Richmond Hill, ON, Canada) at −80 °C. The human resistance data were provided by the Hungarian National Centre for Public Health and Pharmacy.

For each sample, information regarding the source organ (trachea, cloaca) and the location from which the sample was obtained was known, and the isolates were classified into one of Hungary’s seven administrative regions based on the location. This regional classification allowed for comparison with human resistance data.

### 4.2. Determination of Minimum Inhibitory Concentration (MIC)

The phenotypic expression of AMR was determined by establishing the minimum inhibitory concentration (MIC) values for each bacterial strain according to the methodology of the Clinical Laboratory Standard Institute (CLSI) [55]. The breakpoints were also determined following CLSI guidelines [80] and the results were compared with the epidemiological cut-off values (ECOFF) defined by the European Committee on Antimicrobial Susceptibility Testing (EUCAST). For certain active substances where CLSI breakpoints were not available, we relied on data from the literature, such as for imipenem [75], tylosin [74], and tiamulin [76].

Bacterial strains stored at −80 °C were suspended in 3 mL of cation-adjusted Mueller–Hinton broth (CAMHB), followed by incubation at 37 °C for 18–24 h. The tests were performed using 96-well microtiter plates (VWR International, LLC., Debrecen, Hungary). Except for the first column, all wells were filled with 90 µL of CAMHB. The preparation of 1024 µg/mL stock solutions of the tested substances (Merck KGaA, Darmstadt, Germany) was performed according to CLSI guidelines [80]. The active ingredients, amoxicillin and amoxicillin–clavulanic acid in a 2:1 ratio (pH 7.2, 0.01 mol/L) and imipenem (pH 6, 0.1 mol/L), were dissolved in phosphate-buffer solution. Doxycycline, ceftriaxone, spectinomycin, lincomycin, colistin, neomycin, tylosin, and vancomycin were dissolved in distilled water. For the preparation of the potentiated sulfonamide (trimethoprim and sulfamethoxazole at a 1:19 ratio), sulfamethoxazole was dissolved in hot water with a few drops of 2.5 mol/L NaOH, while trimethoprim was dissolved in distilled water with 0.05 mol/L HCl. Enrofloxacin was prepared using a few drops of 1 mol/L NaOH solution in distilled water. Florfenicol was dissolved using a few drops of 95% ethanol and distilled water. From the 512 µg/mL solution, which was diluted 1:1 with broth, 180 µL was dispensed into the first column of the working plates, followed by a twofold serial dilution. After the 10th column, the excess 90 µL of solution was discarded, leaving 90 µL in each well. A bacterial suspension adjusted to 0.5 McFarland using a nephelometer (ThermoFisher Scientific, Budapest, Hungary) was inoculated into the microtiter plates starting from the 11th column backward, at a volume of 10 µL/well [80]. The evaluation was performed using the Sensititre™ SWIN™ automatic MIC reader (ThermoFisher Scientific, Budapest, Hungary) and the VIZION system software version 3.4 (ThermoFisher Scientific, Budapest, Hungary, 2024). The reference isolate used was *S. aureus* (ATCC 23235).

### 4.3. Statistical Analysis

Statistical analysis was performed using R version 4.1.0 [81]. The normality of the data distribution was tested with the Shapiro–Wilk test. Data that did not follow a normal distribution were further analyzed using non-parametric tests. The resistance of each active substance was examined using the Kruskal–Wallis test [82], which does not assume a normal distribution and is suitable for comparing the medians of several sample groups—ideal for analyzing differences across various isolates. A post hoc test was employed to determine specific correlations between groups. Pairwise comparisons were conducted using the Mann–Whitney U test [83] and t-tests, with Bonferroni correction applied to adjust for inflated p-values resulting from multiple comparisons [84]. It is important to note that while the Bonferroni correction reduces the likelihood of Type I errors, it may increase the risk of Type II errors (failure to detect true differences). Further correlation analyses were conducted to explore relationships between individual active substances, followed by principal component analysis (PCA) [85] to identify similarities or differences in patterns. Hierarchical cluster analysis was then performed, with results presented in a dendrogram [86], providing a visual representation of the distances between isolates and the clustering hierarchy. Cluster analysis is a statistical method used to group data points so that those within the same cluster are more similar to each other than to those in different clusters. We applied hierarchical clustering, which allowed for the visualization of dendrograms (tree structures).

Correlation analysis examines the direction and strength of the relationship between variables. A positive correlation occurs when the values of one variable increase in tandem with the values of another variable, and this is considered perfectly positive if the correlation coefficient is +1. Conversely, a negative correlation occurs when the values of one variable increase as the values of another decrease, and this is considered perfectly negative if the correlation coefficient is −. If the coefficient is 0, there is no linear relationship between the variables. The Pearson correlation coefficient is commonly used for correlation analysis when the relationship between variables is linear, and the data are normally distributed. In other cases, such as when relationships are non-linear or the data are not normally distributed, other methods, such as Spearman’s rank correlation coefficient, are applied. In our study, we used the Pearson correlation coefficient.

## 5. Conclusions

Overall, our findings are consistent with results reported in the international literature, reinforcing the importance of continuous monitoring of commensal *Staphylococcus* strains. The more significant differences observed in a few cases can presumably be attributed to variations in antibiotic usage. Regular, repeated surveys are crucial for establishing a system to track temporal trends, which can reveal patterns and provide long-term projections of resistance dynamics. In addition, mapping the genetic background of multidrug-resistant strains is essential for a more precise understanding of resistance mechanisms. As these strains may act as reservoirs of resistance and pose significant biosecurity risks due to their ability to survive in the animal environment, a better understanding of the resistance mechanisms is essential if we are to identify effective countermeasures. The findings underscore the significance of widespread resistance to long-used antibiotics, particularly within the framework of the One Health approach, emphasizing the need for collaborative efforts between the veterinary and public health sectors. Future research must place greater emphasis on genetic studies, which can provide a more comprehensive understanding of the complexity of antimicrobial resistance. Monitoring antibiotic usage and comparing findings from veterinary isolates with human resistance data are critical steps in tracking the development of antibiotic resistance and implementing the One Health approach, bridging the gap between animal and human health.

## Figures and Tables

**Table 1:** Statistical analysis of the relationship between the sample source and the level of resistance.

Antibiotics	Respiratory–Cloaca Comparation
	*p*-Values
Doxycycline	>0.0001 *
Enrofloxacin	0.4387
^1^ Potentiated sulfonamide	0.5184
Vancomycin	0.8005
Amoxicillin	0.6334
^2^ Amoxicillin–clavulanic acid	0.6437
Imipenem	0.6401
Tylosin	0.5466
Tiamulin	0.1318

* significant difference (*p* < 0.05); ^1^ trimetoprime–sulfametoxazole 1:19 ratio; ^2^ 1:2 ratio.

**Table 2:** Statistical analysis of resistance by utilization type.

Antibiotics	Laying–Broiler	Laying–Breeding	Broiler–Breeding
	*p*-Values		
Doxycycline	0.0336 *	0.8927	0.0730
Enrofloxacin	0.2883	0.0402 *	0.6045
^1^ Potentiated sulfonamide	0.0074 *	0.0109 *	0.9978
Vancomycin	0.5427	0.7159	0.2272
Amoxicillin	0.8889	0.1360	0.1438
^2^ Amoxicillin–clavulanic acid	0.3540	0.6012	0.7879
Imipenem	0.0607	0.6122	0.0233 *
Tylosin	0.0077 *	0.7487	0.0291 *
Tiamulin	0.8055	0.2606	0.1805

* significant difference (*p* < 0.05); ^1^ trimetoprime–sulfametoxazole 1:19 ratio; ^2^ 1:2 ratio.

**Table 3:** Statistical analysis of resistance by age group.

Antibiotics	^3^ Young–^4^ Adult Comparation
	*p*-Values
Doxycycline	0.0058 *
Enrofloxacin	0.6738
^1^ Potentiated sulfonamide	0.0860
Vancomycin	0.3538
Amoxicillin	0.4141
^2^ Amoxicillin–clavulanic acid	0.4741
Imipenem	0.0124 *
Tylosin	0.0026 *
Tiamulin	0.4009

* significant difference (*p* < 0.05); ^1^ trimetoprime–sulfametoxazole 1:19 ratio; ^2^ 1:2 ratio; ^3^ younger than 6 weeks; ^4^ older than 6 weeks.

**Table 4:** Statistical analysis of the relationship between flock size and resistance levels.

Antibiotics	Small–Medium	Small–Large	Medium–Large
	*p*-Values		
Doxycycline	0.1310	0.5647	0.0196 *
Enrofloxacin	0.3589	>0.0001 *	0.0597
^1^ Potentiated sulfonamide	0.6906	0.0019	0.0006 *
Vancomycin	0.0012 *	>0.0001 *	0.0635
Amoxicillin	0.0392 *	>0.0001 *	0.4693
^2^ Amoxicillin–clavulanic acid	0.0552	>0.0001 *	0.1816
Imipenem	0.2127	0.3035	0.6089
Tylosin	0.1331	0.4267	0.3763
Tiamulin	>0.0001 *	>0.0001 *	0.2507

* significant difference (*p* < 0.05); ^1^ trimetoprime–sulfametoxazole 1:19 ratio; ^2^ 1:2 ratio; small—5001–50,000; medium—50,001–100,000; large—>100,001.

**Table 5:** Frequency table of minimum inhibitory concentration (MIC) values obtained for active substances with clinical breakpoints in *Staphylococcus* isolates (*n* = 227) originating from domestic chickens. The upper row for each active substance shows the count, while the lower row displays the percentage. The vertical lines indicate the breakpoints.

Antibiotic	^1^ BP *	0.001	0.002	0.004	0.008	0.016	0.03	0.06	0.125	0.25	0.5	1	2	4	8	16	32	64	128	256	512	1024	MIC_50_	MIC_90_	^2^ ECOFF
	µg/mL																						**µg/mL**		
Amoxicillin	0.5	1	1	1	5	2	9	18	17	40	51	26	10	18	10	3	1	1	1	3	6	3	0.5	8	0.5
		0.4%	0.4%	0.4%	2.2%	0.9%	4.0%	7.9%	7.5%	17.6%	22.5%	11.5%	4.4%	7.9%	4.4%	1.3%	0.4%	0.4%	0.4%	1.3%	2.6%	1.3%			
Doxycycline	0.5	2	1	4	12	22	0	7	2	8	18	48	19	16	14	10	14	20	8	0	0	2	1	64	0.5
		0.9%	0.4%	1.8%	5.3%	9.7%	0.0%	3.1%	0.9%	3.5%	7.9%	21.1%	8.4%	7.0%	6.2%	4.4%	6.2%	8.8%	3.5%	0.0%	0.0%	0.9%			
^3^ Amoxicillin–clavulanic acid	1			1	3	8	13	13	19	53	36	31	7	16	16	2	7	2					0.5	4	0.5
				0.4%	1.3%	3.5%	5.7%	5.7%	8.4%	23.3%	15.9%	13.7%	3.1%	7.0%	7.0%	0.9%	3.1%	0.9%							
Tiamulin	4								2	3	1	27	6	14	10	10	44	68	16	9	11	6	32	256	2
									0.9%	1.3%	0.4%	11.9%	2.6%	6.2%	4.4%	4.4%	19.4%	30.0%	7.0%	4.0%	4.8%	2.6%			
Enrofloxacin	4						4	5	19	9	18	12	35	24	14	25	21	26	11	3	1		4	64	0.5
							1.8%	2.2%	8.4%	4.0%	7.9%	5.3%	15.4%	10.6%	6.2%	11.0%	9.3%	11.5%	4.8%	1.3%	0.4%				
^4^ Potentiated sulfonamide	4											1	18	20	31	39	20	24	6	9	14	45	2	64	0.25
												0.4%	7.9%	8.8%	13.7%	17.2%	8.8%	10.6%	2.6%	4.0%	6.2%	19.8%			
Imipenem	8				4	11	20	33	45	34	27	16	7	22	8								0.25	4	0.125
					1.8%	4.8%	8.8%	14.5%	19.8%	15.0%	11.9%	7.0%	3.1%	9.7%	3.5%										
Vancomycin	32	1	0	1	0	2	3	7	10	49	41	59	25	6	1	1	0	1	0	12	6	2	0.5	4	2
		0.4%	0.0%	0.4%	0.0%	0.9%	1.3%	3.1%	4.4%	21.6%	18.1%	26.0%	11.0%	2.6%	0.4%	0.4%	0.0%	0.4%	0.0%	5.3%	2.6%	0.9%			
Tylosin	64	1	0	1	1	0	6	6	2	5	16	88	11	10	13	0	4	2	2	9	14	36	1	1024	2
		0.4%	0.0%	0.4%	0.4%	0.0%	2.6%	2.6%	0.9%	2.2%	7.0%	38.8%	4.8%	4.4%	5.7%	0.0%	1.8%	0.9%	0.9%	4.0%	6.2%	15.9%			

* BP—breakpoint; ^1^ Clinical Laboratory Standard Institute (CLSI); ^2^ epidemiological cut-off value (EUCAST); ^3^ 2:1 ratio; ^4^ trimetophrime-sulfamethoxazole 1:19 ratio.

## Data Availability

The data presented in this study are available from the corresponding author upon reasonable request.

## Supplementary Materials

The following supporting information can be downloaded at https://www.mdpi.com/article/10.3390/antibiotics14010103/s1: Table S1: Frequency table of the minimum inhibitory concentration (MIC) values (µg/mL) for agents without breakpoints in *Staphylococcus* isolates derived from chickens (*n* = 227).
