# Supplementary material for: Antimicrobial Susceptibility Profiles of Commensal Staphylococcus spp. Isolates from Chickens in Hungarian Poultry Farms Between 2022 and 2023
Source: Antibiotics (Basel). 2025 Jan 17;14(1):103. doi: 10.3390/antibiotics14010103 (PMC11763316; doi:10.3390/antibiotics14010103)
Supplement: Supplementary file 1 [file antibiotics-14-00103-s001.zip › antibiotics-3387216-supplementary.pdf]

Supplementary Table S1 Frequency table of the minimum inhibitory concentration (MIC) values (µg/mL) for agents without breakpoints in *Staphylococcus* isolates derived from chickens (*n*=227). The top row for each agent shows the count, while the bottom row shows the percentage.

Antibiotic	0.001	0.002	0.004	0.008	0.016	0.03	0.06	0.125	0.25	0.5	1	2	4	8	16	32	64	128	256	512	1024	MIC_{50}	MIC_{90}	[1]ECOFF
																						µg/mL		
**Ceftriaxone**								3	13	40	25	35	28	34	20	7	4	3	5	3	7	2	32	-
								1.3%	5.7%	17.6%	11.0%	15.4%	12.3%	15.0%	8.8%	3.1%	1.8%	1.3%	2.2%	1.3%	3.1%			
**Neomycin**							1	1	5	9	48	22	15	16	40	37	9	7	10	3	4	8	128	-
							0.4%	0.4%	2.2%	4.0%	21.1%	9.7%	6.6%	7.0%	17.6%	16.3%	4.0%	3.1%	4.4%	1.3%	1.8%			
**Spectinomycin**														4	34	48	35	56	32	9	9	64	256	128
														1.8%	15.0%	21.1%	15.4%	24.7%	14.1%	4.0%	4.0%			
**Florfenicol**										11	4	69	68	45	12	4	5	5	3	1		4	16	8
										4.8%	1.8%	30.4%	30.0%	19.8%	5.3%	1.8%	2.2%	2.2%	1.3%	0.4%				
**Lincomycin**									2	1	13	14	30	42	35	22	14	5	6	19	24	16	1024	2
									0.9%	0.4%	5.7%	6.2%	13.2%	18.5%	15.4%	9.7%	6.2%	2.2%	2.6%	8.4%	10.6%			
**Colistin**														18	5	25	14	45	22	47	51	256	1024	-
														7.9%	2.2%	11.0%	6.2%	19.8%	9.7%	20.7%	22.5%			

[1] Epidemiological cut-off value (EUCAST)
